# Origin and Global Expansion of *Mycobacterium tuberculosis* Complex Lineage 3

**DOI:** 10.3390/genes13060990

**Published:** 2022-05-31

**Authors:** Yassir A. Shuaib, Christian Utpatel, Thomas A. Kohl, Ivan Barilar, Margo Diricks, Nadia Ashraf, Lothar H. Wieler, Glennah Kerubo, Eyob A. Mesfin, Awa Ba Diallo, Sahal Al-Hajoj, Perpetua Ndung’u, Margaret M. Fitzgibbon, Farzam Vaziri, Vitali Sintchenko, Elena Martinez, Sofia O. Viegas, Yang Zhou, Aya Azmy, Khaled Al-Amry, Sylvain Godreuil, Mandira Varma-Basil, Anshika Narang, Solomon Ali, Patrick Beckert, Viola Dreyer, Mwila Kabwe, Matthew Bates, Michael Hoelscher, Andrea Rachow, Andrea Gori, Emmanuel M. Tekwu, Larissa K. Sidze, Assam A. Jean-Paul, Veronique P. Beng, Francine Ntoumi, Matthias Frank, Aissatou Gaye Diallo, Souleymane Mboup, Belay Tessema, Dereje Beyene, Sadiq N. Khan, Roland Diel, Philip Supply, Florian P. Maurer, Harald Hoffmann, Stefan Niemann, Matthias Merker

**Affiliations:** 1 College of Veterinary Medicine, Sudan University of Science and Technology, P.O. Box 204 Hilat Kuku, Khartoum North 13321, Sudan; 2 Molecular and Experimental Mycobacteriology, Research Center Borstel, 23845 Borstel, Germany; cutpatel@fz-borstel.de (C.U.); tkohl@fz-borstel.de (T.A.K.); ibarilar@fz-borstel.de (I.B.); mdiricks@fz-borstel.de (M.D.); nashraf@fz-borstel.de (N.A.); pbeckert@fz-borstel.de (P.B.); vdreyer@fz-borstel.de (V.D.); sniemann@fz-borstel.de (S.N.); 3 German Center for Infection Research, Partner Site Hamburg-Lübeck-Borstel-Riems, 23845 Borstel, Germany; 4 Institute of Microbiology and Epizootics, Freie Universität Berlin, 14163 Berlin, Germany; wielerlh@rki.de; 5 Robert Koch Institute, 13353 Berlin, Germany; 6 Department of Medical Microbiology and Parasitology, School of Medicine, Kenyatta University, Nairobi 43844-00100, Kenya; glenakerubo@gmail.com; 7 Ethiopian Public Health Institute, Addis Ababa 1242, Ethiopia; eyob2001@gmail.com; 8 Mycobacteria Unit, Bacteriology-Virology Laboratory, CHU Aristide Le Dantec, Dakar 11000, Senegal; awa1.diallo@ucad.edu.sn; 9 Institut de Recherche en Santé de Surveillance Epidemiologique et de Formation (IRESSEF), Diamniadio, Dakar 24600, Senegal; aissatou.gaye@iressef.org (A.G.D.); souleymane.mboup@iressef.org (S.M.); 10 King Faisal Specialist Hospital and Research Centre, Riyadh 11211, Saudi Arabia; hajoj@kfshrc.edu.sa; 11 College of Medicine, Al Faisal University, Riyadh 11533, Saudi Arabia; 12 Department of Medical Laboratory Sciences, Jomo Kenyatta University of Agriculture and Technology, Nairobi 62000-00200, Kenya; perpetualndungu@yahoo.com; 13 Irish Mycobacteria Reference Laboratory, St James’ Hospital, D08 NHY1 Dublin, Ireland; mfitzgibbon@stjames.ie; 14 Department of Clinical Microbiology, Trinity College Dublin, St James’s Hospital, D08 NHY1 Dublin, Ireland; 15 Department of Mycobacteriology and Pulmonary Research, Microbiology Research Center (MRC), Pasteur Institute of Iran, Tehran 1316943551, Iran; f_vaziri@pasteur.ac.ir; 16 Institute of Clinical Pathology and Medical Research, NSW Health Pathology, Sydney 2145, Australia; vitali.sintchenko@sydney.edu.au (V.S.); elena.martinez@health.nsw.gov.au (E.M.); 17 Marie Bashir Institute for Infectious Diseases and Biosecurity, University of Sydney, Sydney 2145, Australia; 18 Instituto Nacional de Saúde, P.O. Box 1120, Marracuene 1220, Mozambique; viegas_sofia@hotmail.com; 19 Chinese Center for Disease Control and Prevention, Beijing 102206, China; carolynzy@hotmail.com; 20 Central Public Health Laboratories, Clinical Microbiology Department, Ministry of Health, Cairo 11835, Egypt; ayaazmy@yahoo.com; 21 Department of Microbiology, Faculty of Veterinary Medicine, Cairo University, Giza 12211, Egypt; kelamry@daralfarouk.com.eg; 22 Laboratoire de Bactériologie, CHU de Montpellier, MIVEGEC, Université de Montpellier—CNRS-IRD, 34295 Montpellier, France; godreuil@yahoo.fr; 23 Department of Microbiology, Vallabhbhai Patel Chest Institute, University of Delhi, Delhi 110007, India; mandirav2001@yahoo.com (M.V.-B.); anshikanarang@gmail.com (A.N.); 24 St. Paul’s Hospital Millennium Medical College, Addis Ababa 1000, Ethiopia; solomon.ali@ju.edu.et; 25 Department of Pharmacy and Biomedical Science, La Trobe Institute for Molecular Science, La Trobe University, Bendigo 3552, Australia; mwila_kabwe@yahoo.com; 26 School of Life and Environmental Sciences, University of Lincoln, Lincoln LN6 7TS, UK; matthew.bates@ucl.ac.uk; 27 Division of Infectious Diseases and Tropical Medicine, University Hospital, LMU Munich, 80802 Munich, Germany; hoelscher@lrz.uni-muenchen.de (M.H.); rachow@lrz.uni-muenchen.de (A.R.); 28 German Centre for Infection Research (DZIF), Partner Site Munich, 80802 Munich, Germany; 29 Infectious Diseases Unit, Fondazione IRCCS Ca’ Granda, Ospedale Maggiore Policlinico, Centre for Multidisciplinary Research in Health Science (MACH), University of Milan, 20122 Milan, Italy; andrea.gori@unimi.it; 30 Laboratory for TB Research and Pharmacology, Biotechnology Centre of Nkolbisson, University of Yaoundé I, Yaoundé 8698, Cameroon; etekwu@yahoo.fr (E.M.T.); lsidze@yahoo.com (L.K.S.); assamjean@yahoo.fr (A.A.J.-P.); v.penlap@yahoo.fr (V.P.B.); 31 Faculty of Medicine, Institute for Tropical Medicine, University of Tübingen, 72074 Tübingen, Germany; ffntoumi@hotmail.com; 32 Fondation Congolaise Pour la Recherche Médicale (FCRM), Villa D6, Campus WHO-AFRO, Brazzaville 2672, Democratic Republic of the Congo; 33 Allgemeinmedizin, Berliner Ring 39, 72076 Tübingen, Germany; matthias.frank@uni-tuebingen.de; 34 Department of Medical Microbiology, College of Medicine and Health Sciences, University of Gondar, Gondar P.O. Box 196, Ethiopia; bt1488@yahoo.com; 35 Department of Microbial, Cellular and Molecular Biology, Addis Ababa University, Addis Ababa P.O. Box 1176, Ethiopia; dereje.bd@gmail.com; 36 Department of Medical Lab Technology, University of Haripur, Haripur 22621, Pakistan; snkhan@uoh.edu.pk; 37 Institute for Epidemiology, University Medical Hospital Schleswig-Holstein, 24105 Kiel, Germany; roland.diel@epi.uni-kiel.de; 38 Lung Clinic Grosshansdorf, Airway Research Center North (ARCN), German Center for Lung Research (DZL), 22927 Großhansdorf, Germany; 39 CNRS, INSERM, CHU Lille, U1019-UMR 9017-CIIL-Center for Infection and Immunity of Lille, Institut Pasteur de Lille, Université de Lille, 59000 Lille, France; philip.supply@ibl.cnrs.fr; 40 National and WHO Supranational Reference Center for Mycobacteria, Research Center Borstel, Leibniz Lung Center, 23845 Borstel, Germany; fmaurer@fz-borstel.de; 41 Institute of Medical Microbiology, Virology and Hygiene, University Medical Center Hamburg-Eppendorf, 20251 Hamburg, Germany; 42 Institute of Microbiology and Laboratory Medicine (IML), WHO/GLI-Supranational Reference Laboratory of Tuberculosis, Robert-Koch-Allee 2, 82131 Gauting, Germany; h.hoffmann@imlred.de; 43 SYNLAB Gauting, SYNLAB MVZ Dachau, 82131 Gauting, Germany; 44 Evolution of the Resistome, Research Center Borstel, 23845 Borstel, Germany

**Keywords:** *Mycobacterium tuberculosis*, MTBC, Lineage 3, back to Africa

## Abstract

**Simple Summary:**

Tuberculosis still causes 1.5 million deaths annually and is mainly caused by *Mycobacterium tuberculosis* complex strains belonging to three evolutionary modern lineages (Lineages 2–4). While Lineage 2 and Lineage 4 virtually conquered the world, Lineage 3 is particularly successful in Northern and Eastern Africa, as well as in Southern Asia, the suspected evolutionary origin of these strains. Here, we sought to understand how Lineage 3 strains came to the African continent. To this end, we performed routine genotyping to characterize over 2500 clinical isolates from 38 countries. We then selected a representative collection of 373 isolates for a whole-genome analysis and a modeling approach to infer the geographic origin of different sublineages. In fact, the origin of Lineage 3 could be located in India, and we found evidence for independent introductions of four distinct sublineages into North/East Africa, in line with known ancient exchanges and migrations between both world regions. Our study illustrates that the evolutionary history of humans and their pathogens are closely connected and further provides a systematic understanding of the genomic diversity of Lineage 3, which could be important for the development of new tuberculosis vaccines or new therapeutics.

**Abstract:**

*Mycobacterium tuberculosis* complex (MTBC) Lineage 3 (L3) strains are abundant in world regions with the highest tuberculosis burden. To investigate the population structure and the global diversity of this major lineage, we analyzed a dataset comprising 2682 L3 strains from 38 countries over 5 continents, by employing 24-loci mycobacterial interspersed repetitive unit-variable number of tandem repeats genotyping (MIRU-VNTR) and drug susceptibility testing. We further combined whole-genome sequencing (WGS) and phylogeographic analysis for 373 strains representing the global L3 genetic diversity. Ancestral state reconstruction confirmed that the origin of L3 strains is located in Southern Asia and further revealed multiple independent introduction events into North-East and East Africa. This study provides a systematic understanding of the global diversity of L3 strains and reports phylogenetic variations that could inform clinical trials which evaluate the effectivity of new drugs/regimens or vaccine candidates.

## 1. Introduction

With nearly 10 million incident cases and 1.5 million deaths worldwide in 2020, tuberculosis (TB) remains the leading cause of death among humans due to a single pathogen [1]. The vast majority of new TB cases each year occur in South-East Asia (43%), Africa (25%), and the Western Pacific (18%), posing a significant public health risk to the affected countries and health care systems [1]. As an additional challenge for TB control, in 2020, nearly half a million cases of rifampicin (RMP)-resistant (RR) TB were reported, out of which 78% were multidrug resistant (at least isoniazid (INH) and RMP resistant) [1].

Bacteria of the *Mycobacterium tuberculosis* complex (MTBC), the causative agents of TB, are classified into eight human-adapted (L1–L9) and animal-adapted phylogenetic lineages [2,3,4,5]. Globally, strains of L2, L3, and L4 are the most widely spread [6,7,8]. The epidemic success of L2 strains in Eurasia has been associated with drug resistance and hypervirulence, whereas L4 strains appear to be composed of ecologically distinct sublineages. These L4 sublineages include geographically widespread generalist clades causing disease in many different human populations, and geographically restricted specialist clades, closely associated with their sympatric host populations [6,7,8,9].

Although L3 strains constitute a major TB burden for high-incidence regions in South Asia, as well as North and East Africa, and are also potential drivers of the MDR TB epidemic in some parts of the world [2,3,10,11,12,13,14], a clear understanding of the population structure of this main MTBC lineage is lacking. Previous studies suggested an origin for L3 strains in South Asia, followed by dispersal to North and East Africa, likely via close economic and cultural interactions between South-East Asia and East Africa in the Common Era [10,15]. However, no study is available that combines a comprehensive phylogenetic framework with phylogeographic inference to provide robust insights into the genetic background and global spread of L3 strains.

To address this knowledge gap, we leveraged a collection of 2682 clinical L3 strains from 38 countries to define the global population structure and phylogenetic roots of L3 strains. We further investigated the geographical dispersal of L3 strains and determined their potential associations with drug-resistant TB.

## 2. Results

### 2.1. Global Population Structure of MTBC L3 Strains

In this study, we analyzed a total of 2682 clinical L3 strains (one per patient) from 38 different countries, of which 25.9% (*n* = 695) originate from 16 countries in Africa, 32.8% (*n* = 881) from 12 countries in Asia, 18.9% (*n* = 506) from 8 countries in Europe, 12.6% (*n* = 337) from Canada, and 9.8% (*n* = 263) from Australia (Supplementary Table S1). Inclusion criteria were a 24-loci MIRU-VNTR pattern classified as L3 (i.e., Delhi/CAS) using the MIRU-VNTR nomenclature at http://www.miru-vntrplus.org, accessed on 6 August 2020. Overall, 63.8% (1710/2682) of the L3 strains analyzed were either from population-based or cross-sectional studies, of which 1036/2,682 (38.6% overall) had phenotypic drug susceptibility testing (pDST) data available (Supplementary Table S1).

We first constructed a minimum spanning (MS) tree based on 24-loci MIRU-VNTR data of 2682 clinical strains to define major L3 clonal complexes (L3-CC), assigned a nomenclature code (MLVA MTBC 15–9), and estimated the clustering rate among identified L3-CCs (i.e., proportion of strains with identical genotypes) [16,17].

**Figure 1:**
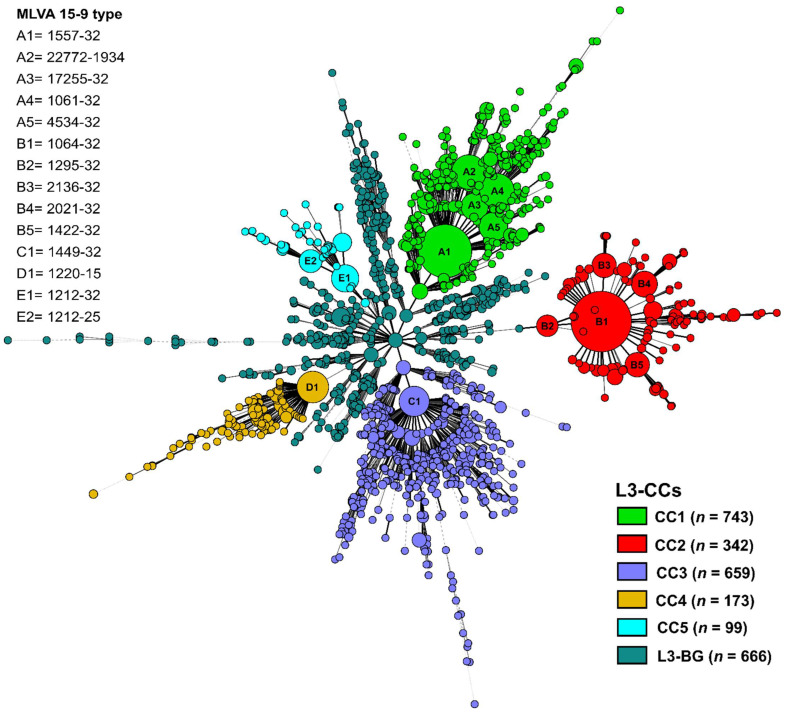
Clonal complexes (CCs) of MTBC Lineage 3 strains. Minimum spanning (MS) tree based on 24-loci MIRU-VNTR data of 2682 clinical MTBC L3 strains originating from 38 countries. Five L3 clonal complexes (L3-CCs) and diverse strains representing the L3 background diversity (L3-BG) are color coded. Node size reflects the number of strains with identical 24-loci MIRU-VNTR patterns. Branch length proportional to the number of allele differences between two nodes. Solid lines indicate 1, 2, or 3 allele differences, gray dashed lines represent 4 allele differences, and gray dotted lines represent 5 or more allele differences.

In total, we identified 1596 different MLVA 15-9 codes, of which 1322 were assigned to a single strain only (Supplementary Table S1). Overall, we identified 1206 strains in 274 clusters, with cluster sizes varying from 2 to 103 strains, including the largest cluster in each of the five L3-CCs: A1 (*n* = 78), B1 (*n* = 103), C1 (*n* = 25), D1 (*n* = 29), E1 (*n* = 21) (Figure 1). We then defined L3-CCs based on the topology of the MS tree, which reveals the structure of relatedness of genotyping patterns by minimizing the weights of the edges between genotypes (Figure 1). Each of the five major above-defined L3-CCs appears as one parental node surrounded by successive layers of descending genotypes. Genotype groups showing a more scattered distribution in the MS tree and lacking a clearly visible structure likely represent the genetic background of L3 strains and were not further assigned to a clonal complex and in the following are termed L3-BG, i.e., L3 genetic background (Figure 1).

**Figure 2:**
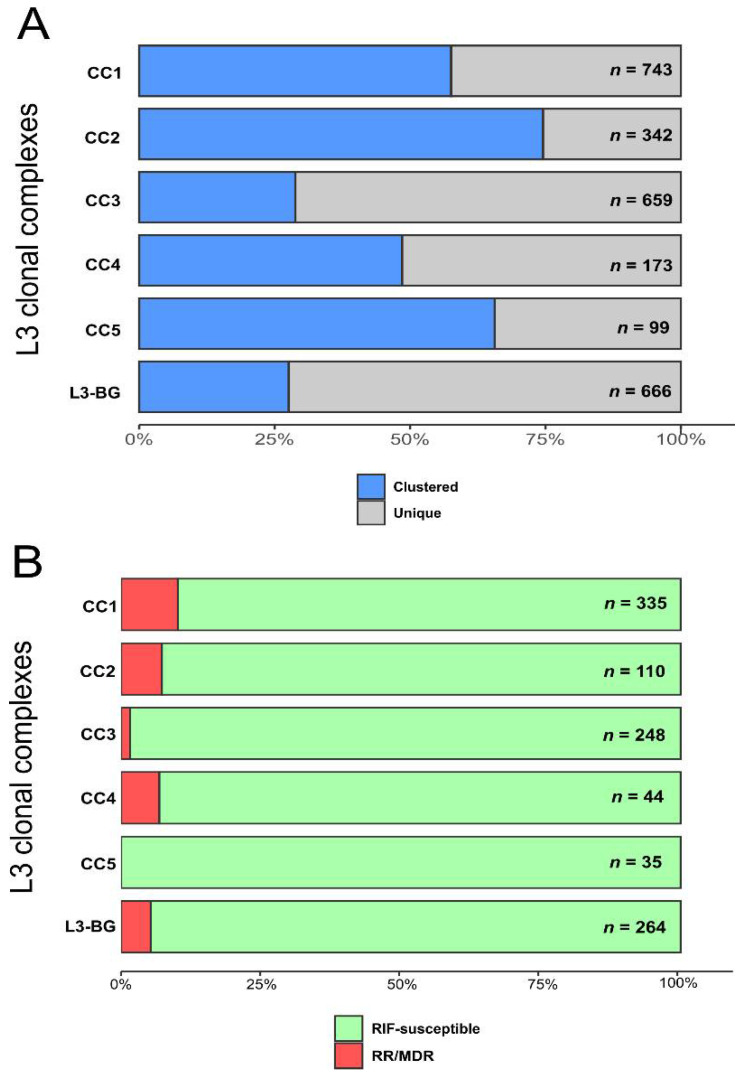
Molecular clusters and drug resistance among L3 clonal complexes (CC). Proportions of clustered strains (**A**) and rifampicin-resistant (RR) and multidrug-resistant (MDR) strains (**B**) within L3 clonal complexes (CC) and within diverse strains representing the genetic background (L3-BG).

Stratified to the five L3-CCs, the proportions of clustered strains (i.e., two or more MTBC strains from different patients and exhibiting identical genotyping patterns [17]) ranged from 28.8% (190/659) in L3-CC3 to 7.6% (255/342) in L3-CC2 (Figure 2a). Strains of L3-CC1, L3-CC4, and L3-CC5 showed intermediate proportions of clustering rates, with L3-CC3 exhibiting the lowest proportion of clustered strains (Fisher’s exact test *p* < 0.001).

We further considered pDST data for strains derived from population-based or cross-sectional studies (1036/2682, 38.6%) and with available pDST results for INH and RIF. Of these, 93.9% (973/1,036) were RIF susceptible, and 6.1% (63/1036) were either RR or MDR. Proportions of RR/MDR strains among L3-CC1 to L3-CC5 were as follows: 10.1% (34/335), 7.3% (8/110), 1.6% (4/248), 6.8% (3/44), and 0.0% (0/35), respectively, with no significant differences (Fisher’s exact test *p* ≥ 0.059) (Figure 2b, Supplementary Table S1). Half (*n* = 31) of the RR/MDR strains occurred in three countries in Northern and Eastern Africa, namely Ethiopia, Kenya, and Sudan.

**Figure 3:**
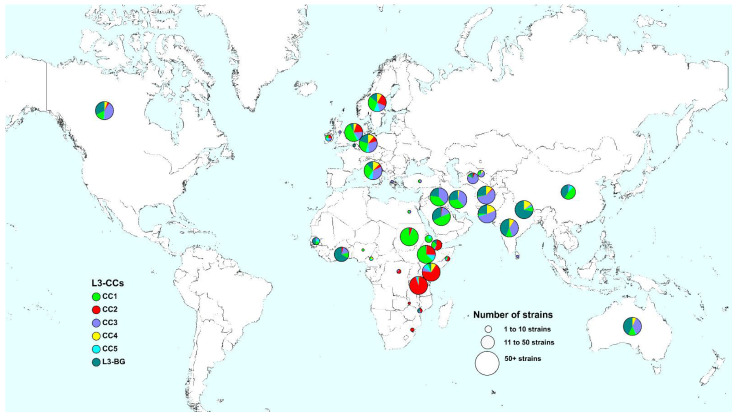
Global geographical distribution of MTBC Lineage 3 clonal complexes (L3-CCs). Each pie chart represents a country, and the size of each pie chart is proportional to the number of strains. L3-CCs and L3-BG are color coded as in Figure 1.

Out of 274 clusters, 137 (50%) comprised strains that were found in 2 to 12 different countries, suggesting a relatively recent cross-border spread of different L3 strains (Supplementary Table S2). The spatial distribution of L3-CCs shows certain L3-CCs dominating in Southern Asia and Northern and Eastern Africa. While Europe shows a higher strain diversity, Australia and Northern America resemble the distribution of L3-CCs in Southern Asia (Figure 3, Supplementary Table S3). Specifically, we observed high proportions of diverse strains representing L3-BG, as well as L3-CC3, in Southern Asia, namely Afghanistan, India, Pakistan, and Nepal, as well as in Iran, Australia, and Canada. L3-CC1 strains are dominant in Northern Africa and the Arabian Peninsula, whereas L3-CC2 strains are highly prevalent in Eastern Africa. In Ghana and in Senegal, we identified mostly L3-BG strains (Figure 3, Supplementary Table S3).

### 2.2. Genome-Based Phylogeny of MTBC L3

To analyze the global phylogeny and geographical origin of MTBC L3 strains at a higher resolution, we selected 152 representative strains from all L3-CCs and L3-BG for an initial WGS analysis (Supplementary Figure S1, Supplementary Table S4). The selected strains comprised 4.6% (34/743) of L3-CC1, 7.6% (26/342) of L3-CC2, 6.4% (42/659) of L3-CC3, 5.8% (10/173) of L3-CC4, 6.1% (6/99) of L3-CC5, and 5.1% (34/666) L3-BG strains (Supplementary Table S4). To represent the global diversity of the L3 population as best as possible, we included 51 strains from 10 African countries, 84 strains from 10 Asian countries, and 17 strains from Europe, and included five Lineage 2 strains as an outgroup. Next, we calculated a maximum-likelihood (ML) tree based on a concatenated sequence alignment of 12,262 single-nucleotide polymorphisms (SNPs) (Supplementary Figure S2).

In the ML phylogeny, we defined 12 clades that correlated well with the MIRU-VNTR-based L3-CCs and/or country of origin, and we defined signature SNPs specific for all strains of each clade (Supplementary Table S4). We then screened for these signature SNPs in a previously published L3 strain dataset from Napier et al. (*n* = 3406) [18]. Overall, 2916/3406 (85.6%) L3 strains in this dataset could be classified with the signature SNPs defined above, while 490/3406 strains (14.4%) remained unclassified. To further extend the genetic diversity in our L3 reference set and the related WGS-based phylogeny, we combined our initial L3-CC- and country-of-origin-based selection (*n* = 152), with 221 strains from Napier et al., including 107 strains with and 114 strains without signature SNPs, respectively (Supplementary Table S5).

**Figure 4:**
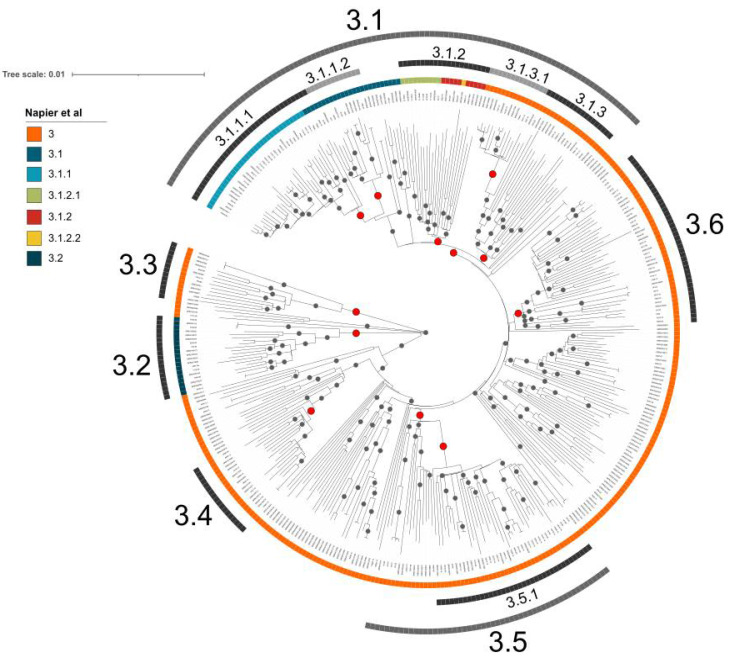
Global phylogeny of MTBC Lineage 3 strains. Midpoint-rooted maximum-likelihood (ML) tree based on 26,536 concatenated single-nucleotide polymorphisms (SNPs) and 373 L3 strains. The first outer ring provides a color code for the L3 subgroups defined by Napier et al. (18). Subsequent outer rings indicate clades for which we determined signature SNPs. Gray and red dots indicate branches with at least 99% bootstrap support; red dots further indicate branches for which we propose signature SNPs.

The resulting ML phylogeny then comprised 373 clinical L3 isolates from 38 countries (Figure 4). The L3 tree topology is characterized by a star-like expansion with short internal and long terminal branches and several multifurcations (i.e., more than two branches descending from one node). The highlighted clades and the overall topology remained stable using Lineage 2 strains as an outgroup (Supplementary Figure S2). There are very few indications for genetic bottleneck events (i.e., long internal branches) in the evolutionary history of L3 strains. The phylogeny is thus rather indicative of an ancient population expansion into different parts of the globe originating from a single common ancestor.

### 2.3. MTBC L3 Origin and Ancestral State Reconstruction

**Figure 5:**
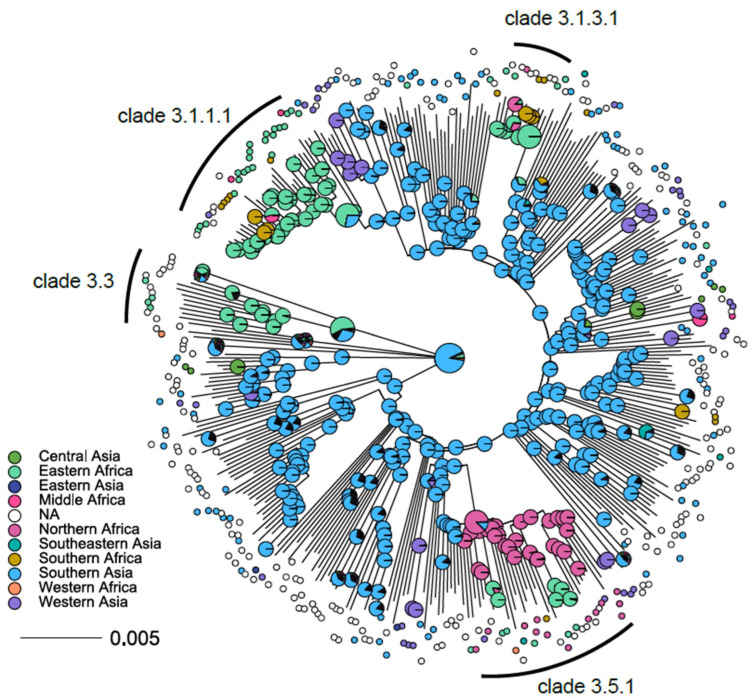
Phylogeographic ancestral state reconstruction of MTBC L3 strains. Nodes are color coded by UN regions. Pie charts show likelihoods for ancestral geographic origins for the internal nodes. A South Asian origin for L3 progenitor is supported using the rooted maximum-likelihood phylogeny. The scale bar indicates nucleotide substitutions per site. Four clades with a predicted African common ancestor are indicated and their pie charts are shown enlarged for better visibility, as well as for the root.

Lastly, we examined with a joint maximum likelihood estimation ancestral state reconstruction of the likely geographical origin of MTBC L3 strains and possible continent transition events of particular L3 clades [19]. As expected, South Asia was predicted to be the origin of the common ancestor of all L3 strains, with a likelihood of 90.2% (Figure 5). However, one of the ancestral clades in the ML phylogeny, Clade 3.3, has a recent common ancestor that most likely originated in Eastern Africa (56.0%, Figure 5). This may suggest a very early introduction of L3 strains into Africa in the evolutionary history of this lineage. The observed geographical split of L3-CC1 and L3-CC2 around the Horn of Africa (Figure 3) is further confirmed by our ancestral state reconstruction. Clade 3.5.1 (associated with L3-CC1) shows a pronounced bottleneck effect in the ML phylogeny and a likely origin in North-East Africa (86.5%), particularly in Sudan (Figure 5). More recent introductions according to the ML phylogeny were observed for Lineage 3.1 strains. Specifically, Clade 3.1.3.1 (associated with L3-CC5), as well as Clade 3.1.1.1 (associated with L3-CC2), have predicted origins in Eastern Africa, with likelihoods of 99.8% and 71.9%, respectively (Figure 5). In summary, we confirm the likely origin of L3 strains in Southern Asia and found evidence of at least four independent introductions of individual L3 clades into East and North-East Africa.

## 3. Discussion

Based on a large-scale analysis of classical genotyping data (24-loci MIRU-VNTR), we developed a first view of the global population structure and spatial distribution of MTBC L3 strains. Whole-genome-based analysis further revealed the global phylogeny and geographical expansion patterns. While we confirm the South Asian origin of L3, we furthermore provide new evidence for at least four independent introduction events into East and North-East Africa in the evolutionary history of this important modern MTBC lineage. Moreover, our findings provide signature SNPs that describe up to 85% of the global L3 genetic diversity, which will be important for clinical trials and pharmaceutical studies that need to take the phylogenetic diversity of MTBC strains into account.

### 3.1. Phylogeography of MTBC L3

Our ancestral state reconstruction confirms that the progenitor of L3 strains can be traced back to South Asia, as suggested earlier, but based on a much smaller, less diverse strain set [20]. Similar to other modern MTBC lineages, the L3 population likely began to expand in parallel with their human hosts and following ancestral movements out of Africa towards Eurasia [20]. The following long-term historic expansion in Southern Asia is reflected by the diverse MIRU-VNTR patterns of strains classified as L3-BG, which are dominating in India and Nepal [3,13].

Interestingly, our phylogeographic analysis further suggests four independent African introduction events of individual L3 clades. The earliest introduction event from Southern Asia into Eastern Africa is suggested for the evolutionarily ancestral clade 3.3 and is consistent with known ancient exchanges and migrations between both world regions. As suggested in a similar scenario for distinct L2 clades [19], three reintroductions occurred more recently, i.e., Clade 3.5.1 (associated with L3-CC1) mainly in Sudan, Clade 3.1.3.1 (associated with L3-CC5), and Clade 3.1.1.1 (associated with L3-CC2) in Kenya and Tanzania. Subsequent strong local expansion of L3-CC1 and L3-CC2 strains is supported by the fact that both clonal complexes also harbor the largest molecular clusters and the geographical separation of CC1 and CC2 around the Horn of Africa and particularly in Ethiopia.

The large geographic distribution of L3 strains beyond South Asia and East Africa likely reflects modern migratory movements. Strains from the largest L3-CC1 cluster (MLVA Code: 1557-32) were retrieved from patients in Australia, Canada, Eritrea, Ethiopia, Germany, India, Italy, Nigeria, Sudan, Sweden, and the Netherlands. Strains of the largest L3-CC2 cluster (MLVA Code: 1064-32) were also identified in 12 different countries in eight UN geo-regions across four continents, including Asia, Africa, Europe, and North America. Since the first half of the 20th century, North America, Europe, and Australia have been connected by extensive movement of workers from low-income countries in Asia and Africa [21,22,23,24,25]. In recent years, for instance, over half of the immigrant labor force of Canada and Australia were born in Asia, with nearly 60% of these immigrants born in China and India [26,27].

### 3.2. Implication of (MTBC and) L3 Genomic Diversity

Despite the strongly clonal population structure of the MTBC, evidence is accumulating on phylogenetically associated biomedical consequences of the diversity of the pathogen. This genomic diversity can potentially lead to different pathobiological features, such as disease severity [28,29] and dormancy [28,30,31], or can affect treatment outcomes [32], bacterial fitness [33], or resistance levels to anti-TB drugs [34]. (Phylo)genetic determinants of such pathobiological differences have recently been pointed out. The loss of the TbD1 genomic region in modern MTBC lineages, comprising L2, L3, and L4, has been recently suggested to be a key driver of their global epidemic spread, as this loss causes an increased resistance against hypoxia, oxidative stress, and enhanced virulence in these strains, relatively to TbD1-intact strains, such as L1 and L7 [35]. Likewise, some phylogeny-linked SNPs in the *esxW* gene or deletion in the *ppe38* gene have been associated with increased transmissibility and hypervirulence of the Beijing lineage (L 2.2) [9,36]. Conversely, mutations in the *phoR* gene shared by the animal-adapted and *M. africanum* L6 lineages have been associated with lower virulence or transmissibility [37]. Here, we identified signature SNPs that were able to differentiate more than 85% of a global L3 strain collection. Our extended L3 nomenclature can be combined with existing SNP barcodes for Mtbc strains [18] and allows a more detailed classification of L3 strains for surveillance or clinical studies. Furthermore, these clade-specific SNPs can be important for the evaluation of phenotypic discrepancies between L3 strains such as response to individual antibiotics or potential new vaccine candidates.

### 3.3. Limitations

Molecular, phenotypic, and geographical data were collated from different studies and diverse sampling methods. Thus, the proportions of L3 strains and sizes of molecular clusters can differ in countries, especially those with only few available datasets. Moreover, to overcome the bias introduced by collections enriched with rifampicin-resistant strains, we only considered cross-sectional and population-based cohorts for the analysis of resistance determinants among clonal complexes.

## 4. Conclusions

Our findings extend the knowledge of the global genetic diversity of MTBC L3 strains. By employing large-scale 24-loci MIRU-VNTR data and WGS-based phylogeographic analysis, we offered a robust explanation of how L3 strains were independently introduced into North and East Africa and into other regions of the world. The evolutionary success of this important modern MTBC lineage is likely shaped by ancient cultural exchange and modern labor migrations from Africa and South Asia to Australia, North America, and Central Europe. The identified SNP signatures reveal an intriguing genetic diversity of L3 strains that needs to be considered in clinical trials that evaluate the effectivity of new drugs/regimens or vaccine candidates.

## 5. Materials and Methods

### 5.1. Data Collection

Our study is based on a global collection of 2682 clinical MTBC L3 strains from 38 countries. For patients in Western Europe with a migration background, we used country of birth as a surrogate. Datasets were compiled from 53 studies. Details on sampling time, sampling strategies, drug susceptibility patterns, and 24-loci MIRU-VNTR data with MLVA 15–9 genotypes and associated cluster numbers are presented in Supplementary Table S1.

### 5.2. 24-Loci MIRU-VNTR Typing

The 24-loci MIRU-VNTR typing was conducted according to standardized protocols published earlier [38]. Briefly, MIRU-VNTR alleles were amplified using the Quadruplex PCR Kit (Genoscreen, Lille, France) according to the manufacturer’s instructions. Fragment analysis using the GeneScan™ 1200 LIZ dye as a size standard (Life Technologies, Darmstadt, Germany) was carried out on a capillary sequencer 3130*xL* and 3500*xL* for the genetic analyzer. GeneMapper software version 3.7 (Life Technologies, Darmstadt, Germany) was used to determine the copy number of MIRU-VNTR alleles. We considered strains with a maximum of one missing locus and classified them as L3 (Delhi/CAS genotype) by phylogenetic inference from a reference collection hosted at http://www.miru-vntrplus.org, accessed on 06 August 2020 [16]. Further, we assigned MLVA MTBC 15-9 codes for strains with complete MIRU-VNTR profiles. A minimum spanning (MS) tree was calculated based on 24-loci MIRU-VNTR typing data (a missing locus was treated as its own category) with BioNumerics v7.6 (Applied Maths (bioMérieux), Sint-Martens-Latem, Belgium) [39]. Strains with identical MLVA MTBC 15-9 codes were combined in a molecular cluster, as a surrogate marker for recently transmitted strains. Based on the MS tree topology, we selected major nodes that were considered as parental nodes for globally expanding L3 strain populations, i.e., L3 clonal complexes (L3-CCs). Strains descending from the parental nodes (multilocus variants) were assigned to the respective L3 CCs. The remaining strains which did not show a clear structure were considered as having a diverse L3 genetic background (L3-BG).

For whole-genome sequencing (see below), we selected strains from the largest nodes and associated multilocus variants from the MS tree (Supplementary Figure S2). Selection criteria were equal representation of all defined L3-CCs and L3-BG, inclusion of different countries, and availability of cultures/DNA at the Research Center Borstel in Germany.

### 5.3. Whole-Genome Sequencing Analysis

WGS was performed with Illumina (San Diego, CA, USA) technology and Nextera XT library preparation [40], as instructed by the manufacturer. Strains were sequenced with a minimum average genome coverage of 50x. Raw read data were processed with MTBSeq [41] and mapped to the *M. tuberculosis* H37Rv genome (GenBank ID: NC_000962.3). Briefly, variants were called if supported by at least four reads in forward and four reads in reverse orientation, and four reads calling the allele with at least a Phred score of ≥30 and a minimum allele frequency of 75%.

For phylogenetic analysis, identified single-nucleotide polymorphism (SNP) positions were combined into a concatenated sequence alignment, complemented with data from original mappings where necessary [42], excluding positions without a nucleotide call and positions that failed in the above-mentioned criteria for variant calling in more than 5% of the samples. Likewise, repetitive regions (e.g., PPE/PGRS genes) and drug-resistance-associated genes were excluded from the phylogenetic reconstruction [42].

### 5.4. Phylogenetic Analysis

Maximum-likelihood (ML) trees were calculated with FastTree using concatenated sequence alignment and a general time-reversible (GTR) substitution model [43]. Inspection and midpoint rooting of the ML tree were performed with FigTree v1.4.4 software, while graphical annotation was performed with the online tool EvolView [44] and iTol [45]. Major phylogenetic clades were defined on the basis of the tree topology, 100% bootstrap support, a previously defined L3 sublineage nomenclature [46], and considering L3-CCs based on 24-loci MIRU-VNTR data (see above) [16]. Clade-specific SNPs were extracted from the concatenated sequence alignment. WGS datasets were further inspected for the presence of the L3-specific deletion RD750 using Integrative Genomics Viewer (IGV) [47].

### 5.5. Ancestral State Reconstruction

Geographic origin was used as a discrete state in the ancestral state reconstruction and inferred from patients or sample country of origin and translated to the associated UN region. Origin of samples sequenced in Europe, the United States of America, and Australia lacking any patient information was set to “NA” in order to avoid geographical bias from larger sequencing collaborations. For an ML phylogeny of 373 L3 strains, we selected the best model of character evolution by calculating the Akaike information criterion for an equal-rates (ER) model, a symmetric, unordered model (SYM), and an all-rates-different (ARD) model with fitMk from the R package phytools [48] and computing their Akaike weights. Scaled likelihoods for each ancestral state were calculated with the best fitting model ER (aic.w = 0.96) and the ace function from the R package ape and mapped on the ML phylogeny [49].

### 5.6. Statistics

Differences between proportions of individual groups were calculated with pairwise Fisher’s exact tests.

## Data Availability

Whole-genome sequencing data (fastq files) were uploaded to the European Nucelotide Archive (ENA) under Project Accession Number PRJEB42771. Individual accession numbers are given in Supplementary Table S4.

## Supplementary Materials

The following supporting information can be downloaded at: https://www.mdpi.com/article/10.3390/genes13060990/s1, Table S1: Metadata and 24-loci mycobacterial interspersed repetitive units—variable number tandem repeat results for 2682 *Mycobacterium tuberculosis* Lineage 3 strains; Table S2: Mycobacterium tuberculosis Lineage 3 cross-border clusters; Table S3: Geographical distribution of L3 clonal complexes of 2682 Mycobacterium tuberculosis Lineage 3 strains; Table S4: Signature SNPs for 12 major Mycobacterium tuberculosis Lineage 3 clades; Table S5: Whole-genome sequencing data and accession numbers of 373 Mycobacterium tuberculosis Lineage 3 strains; Figure S1: Minimum spanning tree based on 24-loci mycobacterial interspersed repetitive units—variable number tandem repeat results from 2682 *Mycobacterium tuberculosis* Lineage 3 strains and selected L3 datasets colored in blue for whole-genome sequencing (WGS) and phylogenetic inference; Figure S2: Maximum-likelihood phylogeny of 152 Lineage 3 strains from this study with Lineage 2 strains as outgroup.
