# Supplementary material for: Origin and Global Expansion of Mycobacterium tuberculosis Complex Lineage 3
Source: Genes (Basel). 2022 May 31;13(6):990. doi: 10.3390/genes13060990 (PMC9222951; doi:10.3390/genes13060990)
Supplement: Supplementary file 1 [file genes-13-00990-s001.zip › supplementary-genes-1688304/supplementary figures.pdf]

Supplementary Figure S1

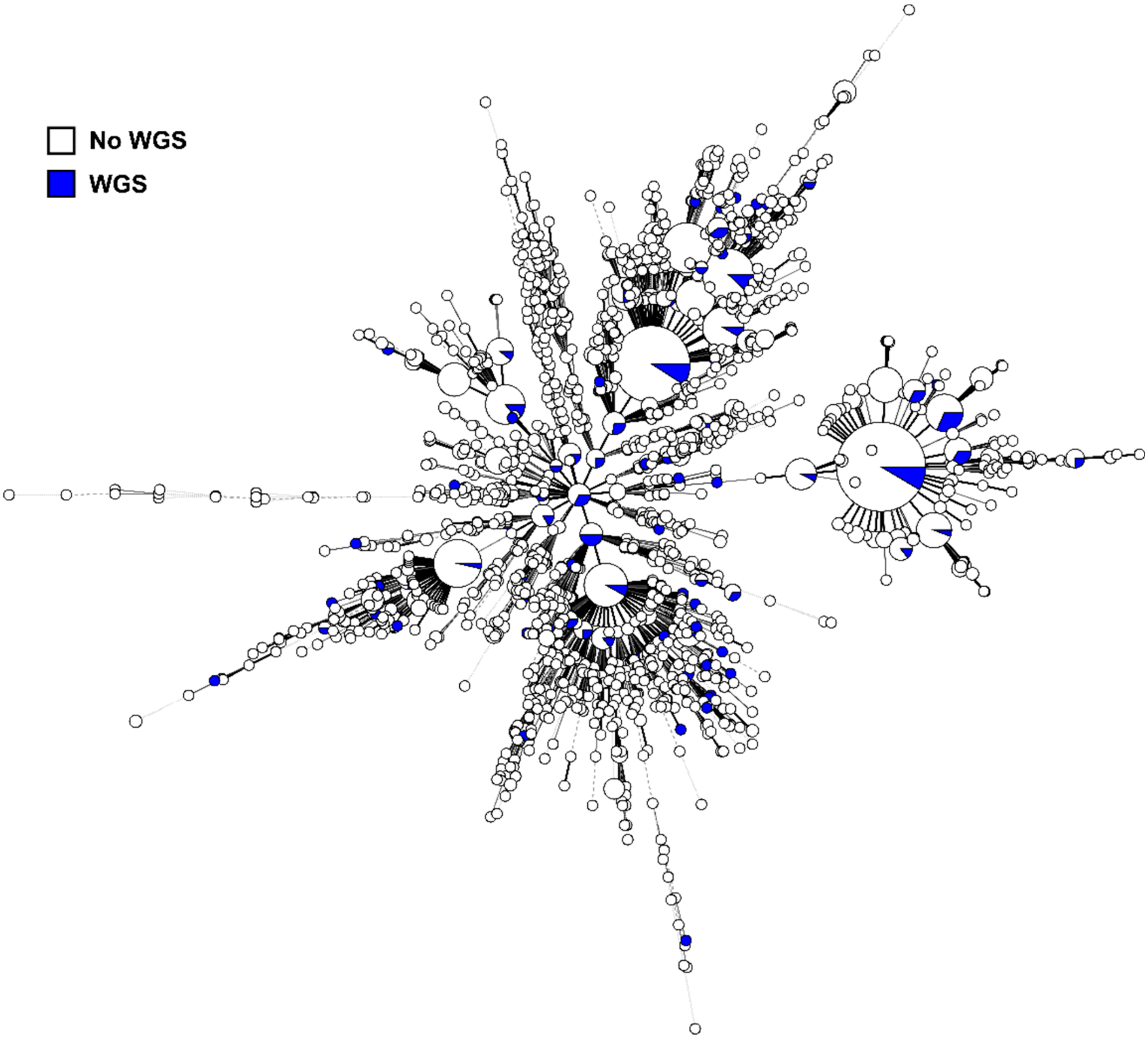

Figure S1. Selected L3 strains for whole genome sequencing (WGS). Minimum spanning (MS) tree based on 24-loci mycobacterial interspersed repetitive units – variable number tandem repeat (MIRU-VNTR) data of 2,682 clinical *Mycobacterium tuberculosis* complex (MTBC) lineage 3 (L3) strains originating from 38 countries. Selected L3 strains for WGS are color coded. Branch length denotes to the number of allele changes between any two strains. Solid lines represent ≤3 allele differences, gray dashed

Supplementary Figure S2

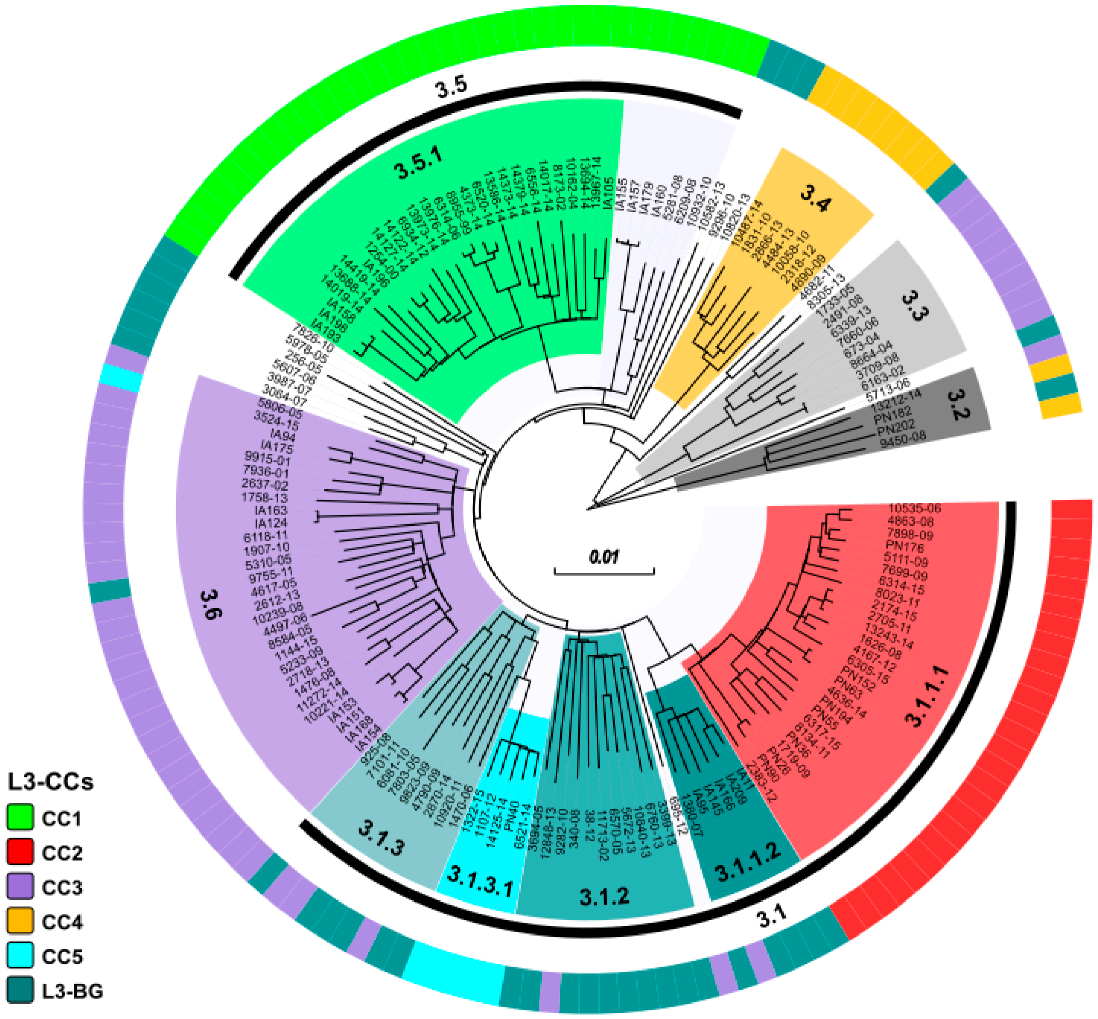

Figure S2. Signature clades of MTBC lineage 3 strains. Midpoint-rooted Maximum likelihood (ML) tree based on 12,262 concatenated single nucleotide polymorphisms (SNPs) and 152 strains originating from 21 countries in Asia, Africa, and Europe. Major clades with 100% bootstrap support are color coded and provided with a tentative nomenclature and signature SNPs extending the lineage barcode from Coll et al (2) and Napier et al (16). The outer ring provides a color code for the L3 clonal complexes based on classical genotyping data (24-loci MIRU-VNTR). The ML tree topology remained the same when ancestral lineage 2 strains were used as outgroup.
