# Supplementary material for: Origin and Global Expansion of Mycobacterium tuberculosis Complex Lineage 3
Source: Genes (Basel). 2022 May 31;13(6):990. doi: 10.3390/genes13060990 (PMC9222951; doi:10.3390/genes13060990)
Supplement: Supplementary file 1 [file genes-13-00990-s001.zip › supplementary-genes-1688304/Supplementary table S2_cross_border_clusters.pdf]

Supplementary Table S2: Clusters of L3 strains reported in at least two different countries, CC=clonal complex, MLVA= Multiple-locus VNTR (variable number tandem repeat) analysis

No of strains	CCs	MLVA code	Cluster ID	Country
2	1	17531-722	2	Germany, Uzbekistan
103	2	1064-32	5	Canada, Eswatini, Ethiopia, Germany, India, Italy, Kenya, Somalia, Sudan, Sweden, Tanzania, Zambia
9	2	4439-32	8	Canada, Eswatini, Kenya, Sweden, Tanzania
2	2	12539-32	9	Ethiopia, Sweden
2	2	1491-32	10	Canada, Kenya
3	2	17651-32	11	Congo, Kenya
19	2	2021-32	12	Canada, Tanzania
3	2	1064-145	13	Ethiopia, Germany
17	2	2136-32	14	Germany, Kenya, The Netherlands
13	2	1295-32	16	Ethiopia, Germany, Ireland, Kenya
3	2	1064-15	17	Italy, Sweden
17	2	1422-32	19	Australia, Congo, Germany, Kenya, Mozambique, Tanzania
2	2	10417-32	22	Canada, Germany
7	2	12794-32	24	Germany, Kenya
2	2	7210-229	25	Eswatini, Mozambique
3	2	1064-25	28	Kenya, Tanzania
3	L3-BG	7800-15	35	Australia, Canada, Iraq
2	1	22824-15	38	Uzbekistan, Germany
3	1	13208-32	40	India, Ireland, Pakistan
2	1	12566-32	41	Iran, Sweden
2	3	4522-32	43	Australia, Canada
2	L3-BG	6783-32	44	Canada, Iran
3	1	12557-32	45	Eritrea, India, Sweden
2	L3-BG	13183-32	46	Canada, Nepal
2	1	1219-32	47	Ethiopia, Germany
2	1	17953-32	48	Canada, Senegal
3	1	4535-32	49	Ethiopia, Canada, Sudan
7	1	4536-32	50	China, Germany, India, Sweden
78	1	1557-32	51	Australia, Canada, Eritrea, Ethiopia, Germany, India, Italy, Nigeria, Sudan, Sweden, The Netherlands
3	1	1062-32	52	Canada, Germany, Sudan
3	1	12087-32	53	Canada, Sudan, Sweden
2	1	9043-32	54	Canada, Ethiopia
4	1	4539-32	58	Canada, Ethiopia, Germany, India
23	1	4534-32	59	Canada, Ethiopia, Germany, Sudan, Sweden
9	1	1557-419	60	Ethiopia, Sudan, Sweden
20	1	17255-32	61	Eritrea, The Netherlands
2	1	1557-25	62	Ethiopia, Sudan
3	1	9259-32	63	Germany, Sudan
4	1	12525-32	67	Sudan, Sweden
2	1	9045-32	69	Egypt, Ethiopia
3	1	15106-32	72	Ethiopia, Sudan
2	1	3658-32	73	Canada, Pakistan
3	1	20770-32	74	Iran, The Netherlands
4	1	12560-32	75	Germany, Sudan
2	1	345-32	78	Ethiopia, Pakistan
2	1	8301-32	79	Canada, Ethiopia
2	1	8301-34	82	Ethiopia, Italy
4	1	4538-32	86	Australia, Germany, Sudan
8	1	1649-32	89	Ethiopia, Germany, Iraq, Sudan
2	1	11471-32	90	Germany, Sweden
2	1	1536-32	91	Ethiopia, Italy

No of strains	CCs	MLVA code	Cluster ID	Country
34	1	1061-32	100	Ethiopia, Germany, Somalia, Sweden
3	1	14680-32	101	Canada, Ethiopia
2	1	9061-32	102	Eritrea, Ethiopia
2	L3-BG	23045-32	103	Canada, Germany
3	1	9773-32	104	Canada, India
2	1	6974-32	108	Sudan, Sweden
5	1	9048-332	109	Eritrea, Ethiopia, Germany
7	3	317-32	114	Australia, Canada, Germany, Pakistan
2	3	3616-32	116	Canada, Pakistan
2	3	3421-32	117	Canada, Pakistan
25	3	1449-32	118	Afghanistan, Australia, Canada, Germany, India, Italy, Pakistan
3	3	3397-32	119	Canada, India
2	3	1473-32	121	Canada, Pakistan
2	3	1449-63	122	Germany, Uzbekistan
4	3	17165-32	123	Australia, Pakistan
2	3	3628-32	127	Egypt, Pakistan
4	3	5085-32	128	Afghanistan, Germany, India, Pakistan
6	3	3612-32	129	Australia, Canada, India
2	3	5847-32	130	Germany, Sweden
2	3	3333-32	133	Australia, Germany
2	3	3635-32	135	India, Pakistan
3	3	3633-32	136	Congo, India, Pakistan
2	3	9756-32	137	Canada, Pakistan
6	2	9072-32	139	Canada, Ethiopia, Germany, Sweden, The Netherlands
2	3	10106-32	140	Afghanistan, Canada
2	3	3629-32	143	Canada, Pakistan
5	3	1320-32	144	Germany, Iran, Pakistan
9	3	3549-145	146	Germany, Iraq
5	3	1534-145	148	India, Italy, Pakistan
5	L3-BG	8966-32	152	Australia, Iraq
3	L3-BG	7814-32	155	Australia, China, India
6	L3-BG	3644-32	156	Australia, Canada, India, Pakistan
4	L3-BG	8738-32	157	Afghanistan, China, Germany, Italy
6	L3-BG	1231-32	159	Afghanistan, Canada, India, Pakistan
2	L3-BG	5758-32	160	Canada, India
2	L3-BG	17366-32	161	Germany, India
2	L3-BG	12549-32	162	Australia, Canada
2	L3-BG	12569-32	163	Germany, Sweden
6	L3-BG	4727-32	164	Afghanistan, Germany, India, Iran, Italy, Pakistan
2	L3-BG	1643-32	165	Afghanistan, India
2	L3-BG	2208-32	166	Canada, Germany
2	L3-BG	2287-32	167	Canada, India
2	L3-BG	9069-32	168	Ethiopia, Pakistan
2	L3-BG	6038-32	169	Australia, India
2	L3-BG	1231-88	170	Australia, India
2	3	21033-32	171	Canada, India
3	L3-BG	17494-32	178	Germany, Uzbekistan, Iran
9	5	12760-32	180	Germany, Sudan
21	5	1212-32	181	Eritrea, Ethiopia, Germany, Ireland, Kenya, Somalia, Sweden, Tanzania
4	5	1242-32	182	Sweden, The Netherlands
3	5	16730-25	184	Canada, Ethiopia

No of strains	CCs	MLVA code	Cluster ID	Country
15	5	1212-25	185	Canada, Ethiopia, Germany, Italy, Senegal, Sweden
3	5	5070-25	186	Italy, Kenya, Sweden
2	5	18539-25	187	Ethiopia, Germany
2	L3-BG	10024-32	194	Afghanistan, Germany
5	L3-BG	6690-32	195	Australia, Germany
4	1	6755-32	197	Eritrea, Ethiopia, Germany
2	3	17409-122	201	Australia, India
2	L3-BG	23555-32	202	Australia, Canada
3	L3-BG	2215-32	203	Canada, Germany, Pakistan
2	L3-BG	19649-32	204	Australia, Germany
8	3	145-62	209	Germany, Iran, Turkmenistan
3	3	1449-162	210	Australia, Canada
2	3	9812-32	235	Afghanistan, Germany
3	3	9714-32	236	Afghanistan, Germany
2	3	3526-32	237	Germany, Iran
2	3	6182-32	238	Australia, Germany
2	3	9635-257	240	Germany, Iraq
3	3	9139-32	241	Turkmenistan, Uzbekistan
2	3	17512-32	243	Germany, Uzbekistan
2	3	9762-32	244	Germany, India
3	1	7186-32	247	Ethiopia, Sudan
2	4	13634-15	252	Canada, Italy
6	4	7122-15	253	Cameroon, Canada, Germany, Kenya, Tanzania
29	4	1220-15	254	Afghanistan, Australia, Canada, Germany, India, Italy, Nepal, Pakistan, Sweden
2	4	3668-15	255	Germany, Pakistan
4	4	3667-15	256	India, Pakistan
2	4	12410-15	257	India, Pakistan
5	4	1719-15	258	India, Italy, The Netherlands
5	4	2218-15	259	Germany, India
2	4	5215-15	260	Australia, Canada
5	4	3354-15	261	Canada, Germany, India, Pakistan
3	4	18513-15	263	Germany, Pakistan
2	4	8540-15	264	Canada, India
2	L3-BG	8854-296	269	Canada, India
2	4	17530-1388	271	Germany, Uzbekistan
