# Supplementary material for: Origin and Global Expansion of Mycobacterium tuberculosis Complex Lineage 3
Source: Genes (Basel). 2022 May 31;13(6):990. doi: 10.3390/genes13060990 (PMC9222951; doi:10.3390/genes13060990)
Supplement: Supplementary file 1 [file genes-13-00990-s001.zip › supplementary-genes-1688304/Supplementary Table S3_geo_distr_2020-09-22.pdf]

Supplementary Table S3: Geographical distribution of individual lineage 3 clonal complexes (CCs) in Asia, Africa, and Europe, BG=genetic background

UN Geo-region	MTBC L3-CCs						Total
	1	2	3	4	5	L3-BG	
**Australia–New Zealand**	35 (4.7%)	1 (0.3%)	92 (14.0%)	18 (10.4%)	4 (4.0%)	113 (17.0%)	**263**
**Central Asia**	3 (0.4%)	1 (0.3%)	18 (2.7%)	1 (0.6%)	0 (0.0%)	3 (0.5%)	**26**
**Eastern Africa**	188 (25.3%)	242 (70.8%)	19 (2.9%)	12 (6.9%)	41 (41.4%)	16 (2.4%)	**518**
**Eastern Asia**	11 (1.5%)	0 (0.0%)	1 (0.2%)	0 (0.0%)	2 (2.0%)	10 (1.5%)	**24**
**Middle Africa**	1 (0.1%)	3 (0.9%)	1 (0.2%)	1 (0.6%)	1 (1.0%)	0 (.0%)	**7**
**Northern Africa**	120 (16.0%)	7 (2.0%)	1 (0.2%)	0 (0.0%)	2 (2.0%)	2 (0.3%)	**132**
**Northern America**	53 (7.1%)	11 (3.2%)	140 (21.2%)	19 (11.0%)	4 (4.0%)	110 (16.5%)	**337**
**Northern Europe**	30 (4.2%)	23 (6.7%)	16 (2.4%)	8 (4.6%)	12 (12.1%)	13 (2.0%)	**102**
**Southern Africa**	0 (0.0%)	3 (0.9%)	0 (0.0%)	0 (0.0%)	0 (0.0%)	0 (0.0%)	**3**
**Southern Asia**	79 (10.6%)	2 (0.6%)	216 (32.8%)	75 (43.4%)	6 (6.1%)	259 (38.9%)	**637**
**Southern Europe**	21 (2.8%)	4 (1.2%)	25 (3.8%)	9 (5.2%)	6 (6.1%)	9 (1.4%)	**74**
**Western Africa**	6 (0.8%)	1 (0.3%)	5 (0.8%)	0 (0.0%)	2 (2.0%)	21 (3.2%)	**35**
**Western Asia**	87 (11.7%)	2 (0.6%)	48 (7.3%)	0 (0.0%)	1 (1.0%)	56 (8.4%)	**194**
**Western Europe**	109 (14.7%)	42 (12.3%)	77 (11.7%)	30 (17.3%)	18 (18.2%)	54 (8.1%)	**330**
**Total**	**743**	**342**	**659**	**173**	**99**	**666**	**2,682**
